# Building workforce capacity to operate a web-based logistics management information system (LMIS) in Pakistan

**DOI:** 10.1186/2052-3211-7-S1-O19

**Published:** 2014-12-17

**Authors:** Muhammad Tariq, Khurram Shahzad, Shyam Lama

**Affiliations:** 1 USAID | DELIVER PROJECT, Islamabad, Pakistan; 2 USAID | DELIVER PROJECT, John Snow Inc., Islamabad, Pakistan

## Background

To improve the availability of contraceptive supplies in Pakistan, the USAID|DELIVER Project worked with the Government of Pakistan (GoP) to develop a national web-based logistics management information system (LMIS), at all tiers of the supply chain. The LMIS automates the collection of data for contraceptives, tuberculosis, and vaccine products. The project also trains users in the skills needed to upload data, and orientates senior staff on the basic aspects of the system to ensure their support.

## Method

To ensure the sustainability of LMIS training, the project developed a cadre of master trainers from government departments at the federal, provincial, and district levels. These trainers monitor the system and conduct roll out training. At each supply chain level, LMIS operators were also chosen to compile and upload data every month. Union council level staff were trained in paper-based reporting. The project provided training manuals, CDs, practical exercises, charts, and job aids to participants.

## Results

The project trained 100 master trainers from all four provinces and three regions of Pakistan. Those master trainers, carefully selected from among qualified GoP staff, trained more than 2,000 LMIS users within health and population welfare departments. These operators enter data from federal, provincial, and district levels and from designated data entry clusters at the sub-district level. The participants’ level of understanding of the LMIS was measured through tests before and after the courses. Results showed significant and satisfactory scores for the majority of trainees. On average, the level of understanding of trainees increased 60-80 percent after the training.

## Discussion

Securing local government commitment to ongoing capacity building and continuous monitoring was a key first step in building the human resources needed for new LMIS. We made a strategic decision to ensure sustainability by selecting the master trainers from within the GoP and developing their capacity to conduct trainings and provide supervision. Consulting with all stakeholders and working with master trainers to create province-specific training plans and materials was also important. Investing in the appropriate individuals from government departments ensured system sustainability and accuracy. Stakeholders now have timely, high quality data upon which to make critical supply chain decisions.

## Lessons learned

Participants’ low level of computer literacy jeopardized the success of the training and the deployment of the LMIS. To remedy this, we added a computer orientation session and instituted on-the-job training and supervision during field visits. We will also provide quality assurance guidance and continued training to master trainers.

